# Oxidative folding in the mitochondrial intermembrane space: A regulated process important for cell physiology and disease

**DOI:** 10.1016/j.bbamcr.2016.03.023

**Published:** 2016-06

**Authors:** Afroditi Chatzi, Phanee Manganas, Kostas Tokatlidis

**Affiliations:** a Institute of Molecular Cell and Systems Biology, College of Medical, Veterinary and Life Sciences, Davidson Building, University of Glasgow, Glasgow, G12 8QQ, UK; b Department of Materials Science and Technology, University of Crete, Heraklion, Crete, Greece; c Institute of Molecular Biology and Biotechnology, Foundation for Research and Technology, Heraklion, Crete, Greece

**Keywords:** Aim, Altered inheritance of mitochondria, ALR, Augmenter of liver regeneration (also called GFER, HPO, HSS), Ccs, Copper chaperone for SOD1, CHCH, coiled coil–helix–coiled coil–helix, CIA, cytosolic iron/sulphur cluster assembly, COX, cytochrome C oxidase, CPC, Cysteine–Proline–Cysteine tripeptide, CytC, cytochrome C, Drp, Dynamin related GTPase protein, Dsb, Disulfide bond formation genes, ER, Endoplasmic reticulum, Ero, Endoplasmic reticulum oxidation gene, Erv, Essential for respiration and viability, FAD, flavin adenine dinucleotide, GFER, Growth factor, Erv1-like protein, GSH, reduced glutathione, GSSG, oxidized glutathione, Hot, Helper of Tim, HPO, hepatopoietin protein, HSP, Heat shock protein, HSS, Hepatic regenerative stimulation substance, IMS, Intermembrane space, ITS, Intermembrane space targeting signal, MIA, Mitochondria import and assembly, MICOS, Mitochondrial contact site and cristae organizing system, MISS, Mitochondrial intermembrane space sorting signal, MPP, Mitochondria processing peptidase, NMR, Nuclear magnetic resonance, PDI, Protein disulfide isomerase, QSOX, Quiescin–sulfhydryl oxidase, SAM, sorting and assembly machinery, SOD, Superoxide dismutase, TIM, Translocase of the inner mitochondrial membrane, TOM, Translocase of the outer mitochondrial membrane, Oxidative protein folding, Mitochondrial protein import, Mitochondrial intermembrane space, Mia40, Erv1, Thiol-disulfide exchange

## Abstract

Mitochondria are fundamental organelles with a complex internal architecture that fulfill important diverse functions including iron–sulfur cluster assembly and cell respiration. Intense work for more than 30 years has identified the key protein import components and the pathways involved in protein targeting and assembly. More recently, oxidative folding has been discovered as one important mechanism for mitochondrial proteostasis whilst several human disorders have been linked to this pathway. We describe the molecular components of this pathway in view of their putative redox regulation and we summarize available evidence on the connections of these pathways to human disorders.

## Highlights

• Mitochondria are the cell center of iron–sulfur cluster assembly and cell respiration.
• The MIA pathway has recently been linked to Fe/S pathways, Ca^2 +^ uptake and apoptosis.
• Mitochondria along with the ER and peroxisomes are major sources of ROS.
• Many diseases have been linked to mitochondrial dysfunction.

## Graphical abstract

**Figure:**
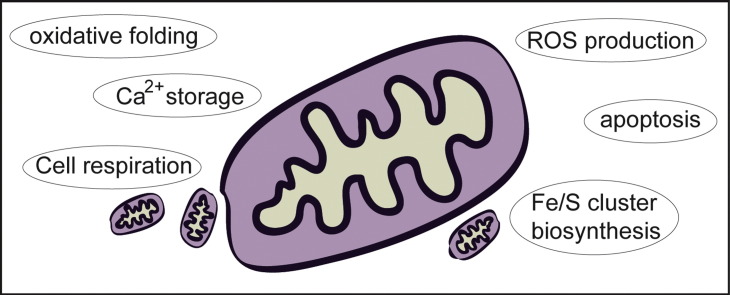

## 1 Introduction

Mitochondria are semi-autonomous cellular organelles that control cellular respiration and metabolism. They have their own set of proteins, which are mostly encoded by nuclear DNA, whilst mtDNA encodes only thirteen proteins. Mitochondrial proteins are sorted within the organelle and are involved in different tasks, whilst the mitochondrial outer and inner membrane differ substantially in their permeability, protein and lipid content, in particular cardiolipin [1]. This particular phospholipid is synthesized in mitochondria, enriched in the inner membrane and mostly found at the two membrane contact points [2], [3]. The presence of the two bilayers leads to the formation of four sub-compartments, each with its own set of functions: the outer membrane, which contains essential proteins of the fission and fusion processes, while acting as a barrier from the rest of the cell; the intermembrane space (IMS), where oxidative folding takes place and factors involved in apoptosis reside [4] ; the inner membrane, which contains the respiratory chain and proteins such as the TIM complexes; and the matrix, where molecules involved in iron–sulfur cluster assembly [5], [6] and the enzymes of the TCA cycle are localized [7].

## 2 Import pathways

The vast majority of mitochondrial preproteins are imported from the cytosol and folded in the organelle, in a process guided by dedicated mitochondrial translocons [8], [9], [10], [11], [12], [13]. Cytosolic chaperones interact with the newly synthesized precursors and prevent their aggregation by protecting their hydrophobic regions from the aqueous environment. The precise targeting of the preproteins is dependent on specific signals in their sequence that typically form an α-helix at the N-terminus with positive charges on one side and are cleaved after import [14], [15], [16], [17]. Not all targeting signals are understood in detail though and there are even internal targeting signals that are capable of leading preproteins to the correct mitochondrial compartment [18], [19], [20], [21]. The cytosolic chaperones transiently interact with the preproteins and deliver them to the receptors of the general entry complex located on the outer membrane, the TOM complex [22], [23]. The interaction of the preprotein-loaded chaperone with the OM receptors exhibits some specificity, but an overlap in the function of one of the OM receptors has been reported [24]. In a very interesting set of recent studies it has been shown that the TOM complex is regulated by phosphorylation events performed by the cytosolic kinases, CK2 and kinase A [24], [25], [26], [27]. Additionally, Tom40 itself has a binding region for precursors allowing it to function independently from the receptors for targeting to the mitochondrial matrix [28], [29]. An additional level of regulation of the import into mitochondria is provided by the thioredoxin and the ubiquitin-proteasome system in the cytosol [30], [31]. The former ensures the reduced state of the preproteins, inhibiting formation of any potential disulfide bridges in the structure of the preproteins prior to import, so that they can be efficiently translocated across the membrane. The latter was shown to degrade unfolded or misfolded preproteins, acting as a negative fine-tuning mechanism dependent on the cellular conditions. In addition to the specific protein-protein interactions along the import pathway, there is evidence that interactions with the lipids of the mitochondrial membranes also play a role in the protein import process, suggesting a coordination for the import and assembly of both proteins and lipids [32], [33], [34].

**Fig. 1:**
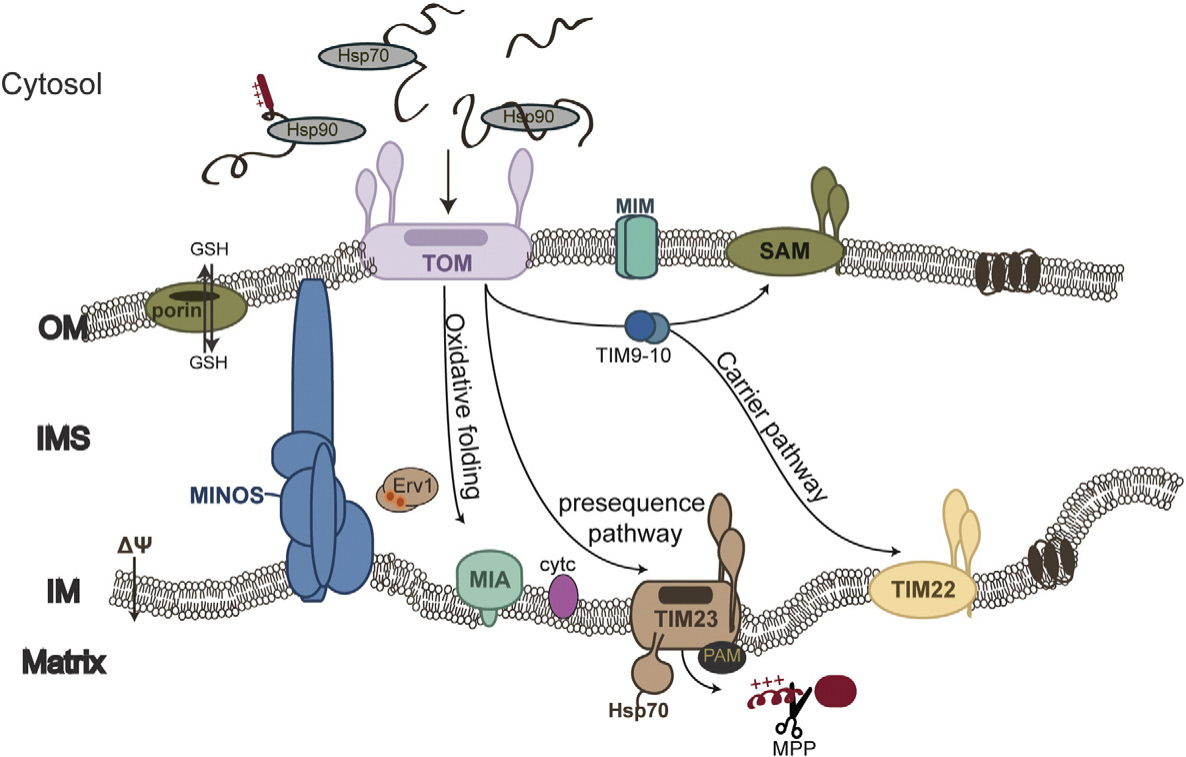
Mitochondrial import pathways. Cytosolic chaperones (Hsp70/90) are responsible for targeting of the precursors to the mitochondrial outer membrane. After interacting with the TOM complex, the preproteins enter the IMS following different sorting pathways (depicted with the different colors) to reach their final destination within the organelle.

The final destination of the precursor proteins determines the specific pathway they will follow (Fig. 1). These different pathways are very elaborate and have been described in detail in several reviews. In short, preproteins targeted to the matrix engage in the TIM23 pathway, the PAM complex and the MPP for their translocation and final folding [9], [16], [35]. Those aimed for integration at the inner membrane usually contain α-helices in their structure and utilize mainly the Tim22 pore. Another subset of inner membrane proteins are integrated via the matrix and the function of the Oxa1 complex [36], whilst β-barrel proteins destined for the outer membrane undergo the SAM pathway [37], [38], [39], [40]. Finally, the intermembrane space hosts proteins that either use the stop-transfer pathway for their release in the IMS (using a variation of the TIM23 pathway that involves specific cleavage of the sorting stop-transfer signal by the dedicated Intermembrane space Protease IMP), or become folded via the MIA oxidative folding system (Fig. 1) [41], [42], [43], [44], [45], [46]. In addition to the large multiprotein translocon complexes, single proteins have been identified in either the IM or the OM with rather specialized functions in the protein import process. This is the case of a small hydrophobic protein, Mgr2 in the IM that functions as a gatekeeper for the TIM23 complex, performing an organelle-specific quality control of precursors prior to their final release in the matrix or the inner membrane depending on their targeting signal [47], [48], [49]. Additionally, the mitochondrial import protein 1 (Mim1) in the OM was shown to play an important role in the import of single-spanning outer membrane proteins [50], [51]. Later studies indicated that Mim1 is critical for the import of multispanning α-helical as well allowing them to integrate more efficiently into the outer membrane [50], [52]. It is therefore thought that Mim1 is part of a distinct import pathway for α-helical proteins into the outer mitochondrial membrane.

In addition to the various dedicated translocon complexes that have been studied in great detail so far, recent efforts have focused on the identification and dynamics of distinct multiprotein complexes that control membrane contact sites either internally in mitochondria or between mitochondria and the endoplasmic reticulum. Four groups [53], [54], [55], [56] have independently identified a large protein complex as an important player in the stabilization of cristae junctions (the internal folds of the mitochondrial inner membrane) which could provide a more efficient scaffold environment for oxidative phosphorylation to occur. This complex was proposed to act as a mitochondrial inner-membrane organizing system (MINOS, or alternatively, MitOS or MICOS) [53], [54], [55], [56] with the commonly agreed name MICOS (*Mi*tochondrial Contact Site and *C*ristae *O*rganizing *S*ystem) [57]. The MICOS complex comprises of six different proteins, of which Mic60 (also known as Fcj1 in *Saccharomyces cerevisiae*) and Mic10 (former name Mio10) are the most important. The maintenance of the cristae junctions by the MICOS complex is essential for the respiratory function of mitochondria, whilst the ATP-generating F_o_F_1_-ATPase preferentially localizes in the cristae [58]. In addition to its critical function in controlling intramitochondrial membrane morphology, the MICOS complex has also been connected to the import pathways since it was shown to interact both with the TOM complex and with the oxidoreductase Mia40, the key component of the MIA pathway [53], [59]. Recently, it was demonstrated that one of the components of the MICOS complex, Mic19, is redox regulated and plays a role in the assembly of the complex and organization of the inner membrane [60].

The molecular analysis of the physical contact sites connecting the mitochondrial and the ER membranes, revealed the presence of the ERMES complex, which is made up by proteins of the outer mitochondrial membrane, the ER protein Mmm1 and a connecting molecule Mdm12, thus bringing together the two organelles [61], [62], [63]. Despite the fact that its role is not yet fully clarified, mutations in the ERMES complex lead to morphological as well as import defects in mitochondria. A proposed model that explains these phenotypes suggests that lipid translocation, between the ER and mitochondria, is coupled to the import of proteins [33], [64]. It is becoming evident that mitochondrial complexes function cooperatively to achieve functional efficiency combining diverse functions, such as protein import, maintenance of membrane integrity and protein folding.

## 3 The MIA pathway

The translocation through the complexes referred to in the above section, as well as the retention of crucial proteins in the IMS via the MIA pathway, is mainly regulated by disulfide bonds [41], [42], [43], [44], [46], [65], [66]. The introduction of these covalent bonds does not only stabilize the proteins in question, but also contributes to their activation.

The chemistry behind this particular mechanism takes advantage of the reduced redox state of the imported precursors and through interactions with oxidoreductases and flow of electrons leads to the introduction of disulfide bonds. Despite the structural differences, this machinery retains its common features in prokaryotes, archaea, as well as specific subcellular compartments of eukaryotic cells, such as the ER and the mitochondrial intermembrane space [67].

In the IMS, the oxidoreductase Mia40 and the sulfhydryl oxidase Erv1 are the key players of the disulfide relay system that facilitate the folding and IMS-retention of the precursors that undergo oxidative folding in this compartment [41], [42], [68], [69], [70]. The role of the disulfide donor in this pathway is ensured by Mia40, a highly conserved protein among eukaryotes. Mia40, also referred to as Tim40, is attached to the inner membrane in yeast, exposing its soluble catalytic domain to the IMS, where it can recognize and bind to specific (‘docking’) cysteines upstream or downstream of the targeting signals (ITS/MISS) of the imported substrates [18], [19]. This binding occurs within a characteristic hydrophobic cleft of Mia40, a structure that is stabilized by two intramolecular disulfide bonds (double CX_9_C motif) [41]. The transient complex between the incoming precursor and Mia40 is held together via a mixed disulfide bond intermediate. The interaction of the precursor with Mia40 leads to the localized folding of the ITS segment within the substrate and the acquisition of the correct intramolecular disulfide bridge between the appropriate cysteine residues of the precursor. The process is completed by the release of the protein in its fully folded state [71], [72]. Next, the dimeric Erv1 interacts with Mia40 to re-oxidize its active CPC motif through thiol-disulfide exchange reactions, which recycles Mia40 to its oxidized state so that it is capable of interacting with another newly imported substrate [73]. The recycling of Erv1 itself is carried out by either cytochrome c that acts as an electron acceptor [68], [69] or by Ccp1 [74]. It is interesting that this well-conserved relay system does not exist in trypanosomatids, a family of protozoan parasites [75], [76]. *In silico* analysis has shown that Mia40 is absent from this organism, in contrast to Erv1 that has a homologue which is thought to take over the entire function of the Mia/Erv1 pathway. However, how this system in *T. brucei* can operate mechanistically is not yet known, although the import of cysteine-rich precursors is dependent on Erv1. Another interesting case is the plant *Arabidopsis thaliana*, where Mia40 is present but non-essential [77]. Deletion of Mia40 results in increased levels of Erv1 in mitochondria, which supports the possibility that Erv1 in this case can functionally replace Mia40, thereby rescuing the Mia40 phenotype.

**Fig. 2:**
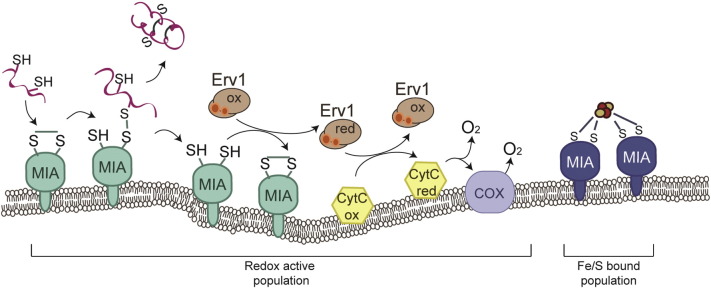
The Mia40 population in the IMS. Mia40 in the IMS functions as an import receptor, a chaperone and as the key oxidoreductase that introduces disulfide bonds to the incoming precursors. In this process, the electrons from the precursor flow to Mia40 then to Erv1 and finally to Cytochrome C. This pathway engages Mia40-redox active population. It has been suggested that a part of the total Mia40 population binds iron/sulfur clusters in a dimer form (Fe/S-bound population). This Mia40 population is considered redox inactive and its function is yet unknown.

In addition to the relay mechanism, an alternative hypothesis suggests that a ternary complex between Mia40, Erv1 and the substrate is crucial for oxidative folding to occur [78]. However, such a ternary complex has not yet been purified and requires further structural and functional study. The fact that Erv1 is sub-stoichiometric to Mia40 would argue that only a fraction of the total Mia40 could be associated in a stable, physical complex with Erv1. On the other hand, the alternative ‘substrate-mimicry’ model was proposed based on interaction and structural data [69]. Erv1 was found to interact with Mia40 in a similar manner as the substrate and on the same hydrophobic cleft, making the concurrent binding to Mia40 an unlikely event. Nevertheless, all available data agree that the oxidative folding pathway is based on the ability of Mia40 to recognize specific motifs in the imported substrates. Such examples are the twin CX_9_C motifs, the twin CX_3_C motifs and the twin CX_2_C motifs (Fig. 2) [18]. Recent work from several groups extended the binding capacity of Mia40 beyond its conventional interaction with small, cysteine-rich IMS proteins. Larger proteins, some even with a co-factor or ligand, with diverse cysteine motifs and no specific docking cysteine were found to rely on Mia40 for their import. The role of Mia40 as a ‘generic’ receptor of the IMS is underpinned by its hydrophobic non-covalent interactions with a variety of precursors that do not necessarily result in the formation of disulfide bonds. One example of a protein without a specific CXnC motif is the mitochondrial protease Atp23, a highly cysteine rich protein, whose import is dependent on Mia40 without a simultaneous oxidation [79]. Atp23 maturation requires multiple rounds of interaction with Mia40. Tim22, a member of the inner membrane translocase that does not have a CXnC motif, was also shown to rely on Mia40 for its import and oxidation, primarily guided by the hydrophobic non-covalent interaction with Mia40 [80].

Recent work by Koch and Schmid [81], [82], [83] has investigated the kinetics of the interaction between Mia40 and its substrates *in vitro*. These studies provided further support for the previous findings that characterized the Mia40 system using structural approaches for the analysis of the interactions *in organello* and in cells. Based on this kinetics analysis, the authors suggested that Mia40 may have an isomerisation function [82]. This is an intriguing possibility which would be important to ascertain *in vivo* and using other substrates in addition to Cox17 that was used *in vitro*. Mia40 and Erv1 themselves depend on the MIA pathway for their biogenesis, but in different ways. Mia40 is imported via the TIM23 complex (N-terminal signal sequence) and depends on the endogenous Mia40 for its subsequent oxidation and folding which is uncoupled from its import. Dissecting the import and assembly of Mia40 in distinct steps, we have shown that folding of the core of Mia40 depends on endogenous Mia40. This step is a prerequisite for the oxidation of its active site CPC motif that depends on the presence of Erv1 [84]. On the other hand, the translocation of the soluble hMia40 (which does not have the N-terminal membrane anchor of the homologous yeast protein) was shown to depend on a targeting signal within its structural core and requires the presence of the human homologue of Erv1 (ALR) [65]. The precursor of Erv1 is imported into the IMS in a Mia40-dependent manner, via the interaction of Mia40 with the structural cysteines of Erv1 located in its C-terminal domain [85]. The flexible N-terminal domain of Erv1 is not required for import but has an auxiliary function in this process.

Recent work in the mammalian system has identified two novel interactors of Mia40/CHCHD4: MICU1, the regulator of the mitochondrial Ca^2 +^ uniporter (MCU) [86], involved in transferring calcium ions across the inner membrane, and AIF1, the apoptosis inducing factor, whose absence is linked to defects of the respiratory chain [87]. The introduction of a disulfide bond between MICU1-MICU2 by Mia40 controls the mitochondrial Ca^2 +^ uptake through association of the heterocomplex with MCU. In the case of AIF1, it was shown that its depletion leads to downregulation of CHCHD4 import that in turn led to respiratory defects.

## 4 A proofreading role for glutathione?

Various studies raise the question of the presence of additional assisting factors in the oxidative folding pathway, such as small molecules or proteins. The IMS-resident protein Hot13 has been proposed to act as a metal binding factor, employed to remove zinc ions from Mia40 to improve its efficiency [88]. The reducing small molecule glutathione has been proposed to serve a proofreading role in the folding of the substrates [89]. In these studies, it was reported that in the presence of low amounts of glutathione, the import efficiency of Mia40-dependent precursors was stimulated, an effect that was similar to that caused by the presence of DTT.

In general, cells employ two major pathways for the redox regulation of the intracellular environment, the glutathione and the thioredoxin pathways [90], [91]. Both of these systems, along with other factors, are essential for controlling and ensuring the action and efficient function of many redox-regulated proteins. It was recently demonstrated that the cytosolic Trx system has the fundamental role of redox regulation, while the mitochondrial matrix GSH pathway overpowers the corresponding Trx couple [92]. As the tripeptide glutathione is synthesized in the cytosol and distributed to all the cell compartments, it is regarded as the main cellular redox regulator due to its high abundance and its prominent role in reducing disulfide bonds.

Taking into consideration its critical role in the network of cellular redox processes, various studies have focused on measuring the levels of GSH:GSSG in the cell. This is a challenging question because the different cell organelles/compartments have different glutathione requirements but also contain different levels of redox-sensitive proteins. Thus, the ratio of GSH:GSSG varies for different organelles. For instance, it has been shown that the cytosol is a reducing environment having a GSH:GSSG ratio of 3300:1, whilst the oxidizing endoplasmic reticulum has a GSH:GSSG ratio of 1:1 to 3:1 [93]. Moreover, the required isolation of the compartment from the rest of the cell could lead to disruption of the balance and probable release of oxidizing amounts of GSH.

Mitochondria pose a particular challenge due to the presence of two distinct intra-organellar compartments: the IMS which is separated by the cytosol from the semipermeable outer membrane, and the matrix, which is surrounded by the impermeable inner membrane. The IMS is only a very small fraction of the whole organelle volume, rendering it very challenging to accurately estimate the two GSH:GSSG pools. In addition, the IMS is further segregated into the intra-cristae lumen and the bulk of the IMS between the outer membrane and the inner boundary membrane. The measurement of the GSH:GSSG ratio from isolated mitochondria varied from 20:1 to 40:1. Nevertheless, a study by Outten et al. in 2008 [94] reported the measurement of the glutathione levels in the IMS and the matrix of mitochondria using *in vivo* redox sensitive YFP sensors. These sensors' cysteines were shown to undergo rapid disulfide exchange reactions only with cytosolic glutaredoxins (GRXs) but not thioredoxins, both *in vivo* and *in vitro*. Specifically, modified rxYFP versions were targeted either to the IMS or the matrix using fusions to appropriate targeting peptides in order to obtain separate measurements of the redox levels. The findings of this study revealed that the IMS is a far more oxidizing environment (GSH:GSSG ration of 250:1) compared to both the matrix (GSH:GSSG ration of 900:1) and the cytosol (GSH:GSSG ration of 3000:1). The result was attributed to the fact that the mitochondrial IMS hosts many proteins that participate to redox pathways, such as members of the respiratory complexes or the MIA pathway for instance.

However, a more recent study by the Riemer group [95], illustrated how the IMS glutathione pool is linked to the cytosolic one via the outer membrane porins. Instead of using the rxYFP, this group used a dynamic redox probe roGFP2 linked with the human Grx1 (Grx1-roGFP2), expressed in the specific compartments. When the cells underwent oxidative stress, the recovery of the corresponding pool was followed through the probe. Glr1 was found to maintain the cytosolic glutathione reduced, while the tripeptide levels were equilibrated with its equivalent in the IMS, rendering it reducing as well. The matrix glutathione pool was unaffected and maintained separately. These findings were developed further by a later study, where it was shown that the IMS hosts limiting levels of cytosolic Grx2 that utilizes glutathione for the redox regulation of Mia40 [96]. These low amounts of Grx2 in the IMS represent an example of the variability that factors affecting the redox measurements, can introduce, given that these probes function through equilibration with glutaredoxins.

The presence in the IMS of a glutathione pool, the localization of some part of cellular Grx2 in this subcompartment and the potential, yet debated, role of Mia40 as an isomerase suggest the presence of a reductive pathway in this sub-organellar compartment. However, a dedicated reductive pathway has not yet been characterized in molecular terms in the IMS. It will be interesting to discover whether such a pathway exists, which factors are functional components of it and whether it shares any functional and/or physical interactions with the oxidative folding pathway.

## 5 ROS regulation

**Fig. 3:**
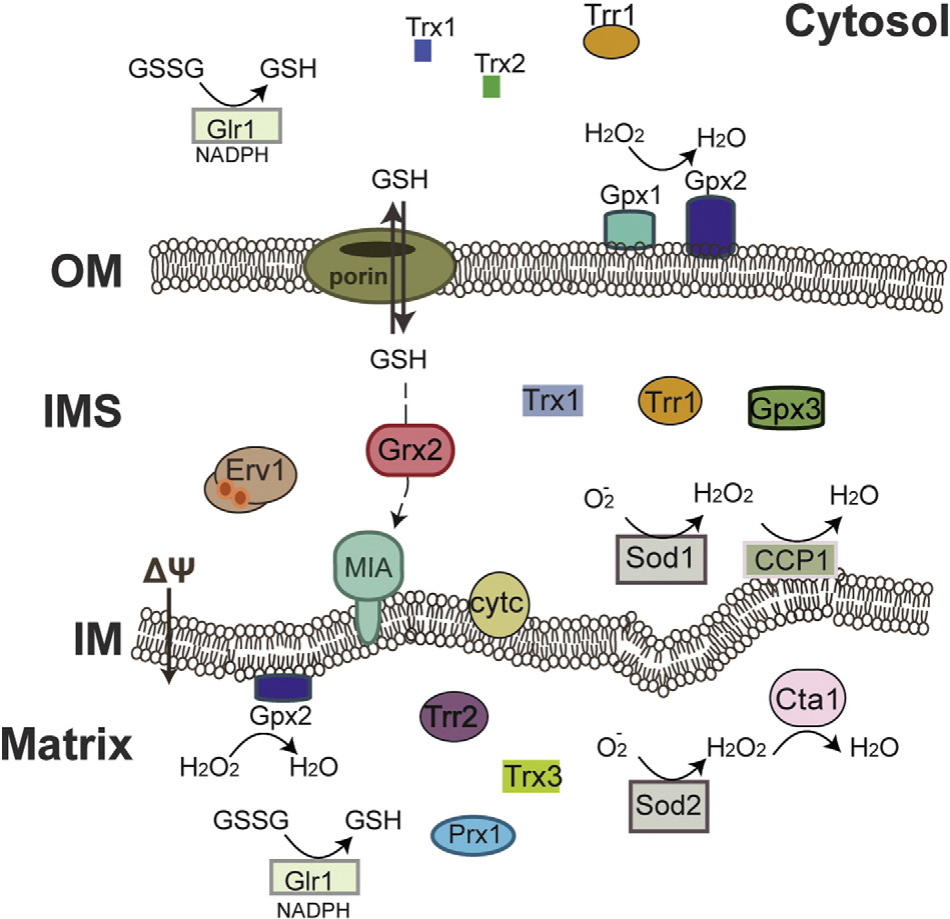
Molecular machineries underpinning redox regulation. The synthesis of glutathione occurs in both the cytosol and the mitochondrial matrix, where there is also a complete Trx system. Other redox regulating protein factors that can be found associated or within mitochondria are Gpx proteins, Grx2, the catalase Cta1 and the SOD proteins. Although functional parallels can be drawn between the cytosolic and mitochondrial systems, the exact nature of the dual localization of some common components and the links to the oxidative folding system remain unknown.

As mentioned above, the thioredoxin and glutathione systems regulate the protein redox state to control both signaling and various cell functions (Fig. 3), but in addition this may be subject to modification by a whole range of different reactive oxygen species (ROS). Mitochondria are major sources of ROS, which are additionally produced by both the ER and the peroxisomes.

In order to keep a balanced level of ROS production, cells have developed a number of protection mechanisms and enzymes, such as the superoxide dismutases (SODs), the peroxidases, the catalases and others [90], [97]. Mitochondria harbor two types of superoxide dismutase enzymes: (i) the copper–zinc binding protein SOD1 which is localized in the IMS of mitochondria (in addition to the cytosol that contains the majority of this enzyme) and (ii) the manganese-SOD2 that is found in the mitochondrial matrix.

Both of these enzymes have a major role in detoxifying the superoxide radicals (Fig. 3). It has been known for a long time that the import and activation of Sod1 depends on the dedicated chaperone protein Ccs1, which in turn is imported following a pathway that depends on Mia40 and the MICOS complex [59]. The activation of the matrix-localized Sod2 on the other hand relies on an interaction with the mitochondrial carrier protein Mtm1 [98].

Another important family of redox controlling enzymes is the glutathione peroxidases one, of which there are three members in the yeast *S. cerevisiae* (Gpx1, Gpx2, Gpx3) and eight isoforms in humans. In yeast cells, Gpx1 is extrinsically associated to the outer membrane of mitochondria [99], [100], [101], where it acts as a phospholipid-hydroperoxide glutathione peroxidase. This is an important function given that lipid peroxidation by ROS affects the permeability of a membrane and thereby its function (Fig. 3). Gpx2 on the other hand is loosely bound to the surface of the mitochondrial inner membrane from the matrix side and has a similar role to Gpx1 [100], [102]. A proteomic analysis of the intermembrane space proteome of *S.cerevisae* mitochondria in 2012 suggested that Gpx3, a Gpx1 paralog, is localized in the IMS of mitochondria [103]. A functional role has not yet been attributed to this mitochondrial association. The majority of Gpx3 is found in the cytosol where it functions as the main hydroperoxide sensor that transduces the oxidative stress signal to Yap1 in the oxidative stress response [100], [104], [105], [106], [107], [108]. Parallels could be drawn between the role of Gpx3 in the cytosol and a putative similar role of the enzyme in the IMS, but the interacting proteins in the IMS and its function in this compartment are completely unknown.

In addition to the Gpx enzymes, yeast mitochondria harbor different forms of glutaredoxin 2 (Grx2) as a system that could have a role in protection against ROS. The cytosolic Grx2 is dually targeted to the IMS, whilst a longer form of Grx2 is targeted to the mitochondrial matrix [109], [110]. This matrix-localized form of Grx2 is thought to play a part in the iron sulfur cluster (ISC) biosynthesis, together with Grx5 a known member of this pathway [111], [112]. The mitochondrial matrix also contains a complete thioredoxin system with Trx3 and its thioredoxin reductase Trr2, being involved in the protection against oxidative stress [113]. Oxidation of Trx3 in the mitochondrial matrix is promoted by the monothiol peroxiredoxin, Prx1, in response to hydrogen peroxide stress to induce programmed cell death (PCD) [114], [115]. Recycling of Prx1 is thought to be mediated by the matrix glutathione pool. In this stress response pathway in the matrix, it is not yet known whether the induction of PCD is directly linked to some conformational change of Trx3 linked to its oxidation or some downstream interaction with other proteins.

The most efficient enzymes in breaking down hydrogen peroxide to water and oxygen are catalases [116], [117]. There are two different catalase isoforms in yeast, one that localizes in the peroxisomes (Ctt1), and one that resides in the cytosol and the mitochondrial matrix (Cta1). mtCta1 is the main detoxification enzyme of the SOD2-produced hydrogen peroxide in the mitochondrial matrix.

## 6 Links to the Fe/S cluster biogenesis pathway

The understanding of the role of glutathione in particular requires further investigation as it is believed that glutathione plays a key role for the operation of the ISC machinery, while also keeping the ROS levels under control.

The mitochondrial matrix is the center of the iron sulfur cluster assembly machinery. The proteins of this pathway interact so that they generate the Fe/S clusters from the cytosolic imported iron, and then export or embed the clusters into matrix-apoproteins [5], [6]. Apart from this machinery, there is also the ISC export apparatus, consisting of the mitochondrial ABC transporter Atm1 of the inner membrane, the FAD binding protein Erv1 and glutathione [73]. Recently, the crystal structure of the pore Atm1 was solved in a complex with glutathione, with a proposed mechanism of glutathione polysulfide export, for iron–sulfur cluster assembly in the cytosol [118], [119]. Some recent studies on Mia40 presented *in vitro* and *in vivo* results demonstrating that Mia40 can also bind to iron/sulfur clusters as a dimer through the catalytic CPC motif, while in mammalian cells its deletion leads to increased iron levels in mitochondria (Fig. 2) [120], [121], [122]. It was pointed out in these reports that the occupation of the active site by Fe/S clusters prevented the interaction with Erv1. However, the role of the population of Mia40 that has an Fe/S cluster bound has not been clarified yet. Another protein that was shown to have a dual localization both in the cytosol and in mitochondria, is the iron sulfur cluster protein Dre2 [123], [124], [125]. It has been shown that Dre2 interacts with Mia40 independently of the presence of Fe/S clusters on Dre2 and this interaction results in the introduction of two disulfide bonds in the Dre2 structure [124]. Later studies followed the localization of Dre2 in more detail and found that it is associated tightly with the outer membrane of mitochondria and in a protease-resistant form [126]. The role of Dre2, either on the mitochondrial membrane or in the IMS has not been determined yet, but one hypothesis would be that its mitochondrial association may be triggered by specific conditions under which the protein has a role in delivery of the iron sulfur clusters from the matrix into the cytosol as part of the ISC export machinery. It would be interesting to test this working hypothesis in future experiments.

## 7 Human diseases

The first human disease that was directly associated with a defect in the mitochondrial protein import pathway was the neurodegenerative Deafness and Dystonia Syndrome [127]. This disorder is caused by a single mutation of C66 to tryptophan in the human homologue of Tim8, a substrate of the oxidoreductase Mia40 [128]. This particular mutation of Tim8 results in the protein remaining in a reduced state unable to undergo proper oxidative folding and incapable of form a complex with its partner Tim13. This causes a multiplicity of mitochondrial defects and leads to a pathological state characterized by deafness and dystonia.

Interestingly, different observations link the oxidative folding pathway in particular to several human disease states. The human homologue of Erv1, called ALR, has been first identified as a key factor modulating liver regeneration after partial hepatectomy (hence its name: *A*ugmenter of *L*iver *R*egeneration). Recently an ALR mutant (R194H) was identified as the cause for the pathological condition of three siblings suffering from developmental delay, hearing loss, progressive muscular hypotonia and congenital cataract [129]. The reported single point mutation affects the protein stability and FAD binding, but does not impair the catalytic function of the enzyme. Another major human disorder that is linked to the Mia40 pathway is ALS (Amyotrophic lateral sclerosis), which is known to be associated with mutations of SOD1 [130], [131], [132], that cannot form the disulfide bonds properly and become aggregated leading to impairment of the mitochondrial function. The import of SOD1 in the IMS relies on the presence of Ccs1 whilst Varabyova et al. [59] showed that Mia40 and the MICOS complex regulate the import of the SOD1 mutants that are linked to ALS. Finally, Brazil et al. demonstrated that glutathione is essential for the activation of hSOD1 independently of the role of Ccs1 in this process [133], [134].

The human homologue of Mia40, CHCHD4, was shown to have different expression patterns in tumor cells and to affect the levels of several IMS proteins [135], [136]. Knockdown of CHCHD4 was correlated with reduced tumor progression and was also linked to the stability of Hif1a, a critical component of the HIF pathway that responds to hypoxia; this was the first report of an association between the mitochondrial disulfide relay system and cancer [136], [137]. Another link of the oxidative folding pathway to cancer was illustrated by the finding that p53, an important tumor control factor that is sensitive to the redox state of the cell, is translocated to mitochondria in a CHCHD4-dependent manner [138]. Overexpression of the oxidoreductase resulted in increased levels of p53 in mitochondria, which impacted on the maintenance of the integrity of mtDNA.

It has been shown that there is a link between the mitochondrial disulfide system and the neurodegenerative Huntington's disorder [139], [140]. This disease is characterized by symptoms such as uncoordinated movement of muscles, depression and stress, in most cases caused by mutations in the Huntingtin gene. The levels of IMS redox proteins that are substrates for the disulfide relay system were altered in a mouse model of Huntington's disease. The majority of the mitochondrial phenotypes observed, depended on the mutated Huntingtin protein levels. Another independent line of evidence linking Mia40 to neurodegeneration was reported by the group of M. Conway. This work showed an increased association between hMia40 and the human branched-chain aminotransferase protein hBCATm, which acts as a redox chaperone controlling protein misfolding and aggregation, a hallmark in Alzheimer's disease) [141].

Although a link between the oxidative folding machinery and the Fe/S cluster biogenesis remains to be shown, it is clear that defects in the mitochondrial Fe/S cluster biosynthesis result in iron accumulation in mitochondria, iron toxicity and enhanced ROS production. Mutations of the protein Frataxin, which is a member of the matrix ISC machinery, have been strongly linked with Friedreich's ataxia [142], [143], [144], [145], excessive iron accumulation in mitochondria and consequently toxicity. The exact mechanism of the interaction of Frataxin with the rest of the ISC proteins remains however to be determined. Another example of a disease related to aberrant levels of iron in mitochondria is the case of erythropoietic protoporphyria which has been linked to mutations of the inner membrane carrier protein mitoferrin 1. Cells of patients suffering from this disease have deficient forms of ferrochelatase, which in some cases was demonstrated to be a direct effect of abnormal expression of mitoferrin1 [146].

## 8 Conclusions

This review aimed to summarize recent progress in the field of mitochondrial protein biogenesis with an emphasis on the oxidative folding system in the intermembrane space, its connection to redox regulation and how this may be linked to several human disorders. In more general terms, understanding the level and mechanistic details of redox regulation in the fundamental processes of mitochondrial proteostasis and iron sulfur cluster biogenesis, can be a turning point in elucidating key determinants of a large group of mitochondria-related human diseases. Future work in this field holds great promise for substantial advances in our understanding of the role of mitochondria in cellular signaling processes both at a fundamental and a translational level.

## Conflict of interest

The authors declare no conflict of interests or any commercial associations.
